# An Intelligent Wearable EMG Sensing Framework for Athlete Neuromuscular Monitoring and Performance Progression Assessment

**DOI:** 10.3390/bios16090457

**Published:** 2026-08-23

**Authors:** Kudratjon Zohirov, Sardor Boykobilov, Gulmira Pardayeva, Nilufar Akhmedova, Dilobar Ilmurodova, Iroda Uralova, Zavqiddin Temirov, Rashid Nasimov

**Affiliations:** 1 Software and Hardware Support of Computer Systems, Karshi State Technical University, Karshi 180100, Uzbekistan; qzohirov@kstu.uz (K.Z.); n.axmedova@kstu.uz (N.A.); 2 Information Technologies, University of Information Technologies and Management, Karshi 180100, Uzbekistan; g.pardayeva@atmu.uz; 3 Department of Information Systems and Technologies, Karshi State Technical University, Karshi 180100, Uzbekistan; dilmurodova@kstu.uz; 4 Department of Informatics and Computer Graphics, Tashkent State Transport University, Tashkent 100167, Uzbekistan; i.abduvaliyevna@tuit.uz; 5 Department of Convergency of Digital Technologies, Tashkent University of Information Technologies Named After Muhammad al-Khwarizmi, Tashkent 100084, Uzbekistan; 6 Department of Digital Technologies, Alfraganus University, Tashkent 100190, Uzbekistan; z.temirov@afu.uz; 7 Department of Computer Engineering, Gachon University, Sujeong-gu, Seongnam-si 461-701, Republic of Korea

**Keywords:** EMG sensing, muscle activity detection, athlete monitoring, wearable sensing systems, regression-based prediction

## Abstract

Electromyography (EMG)-based sensing is an important tool for assessing neuromuscular activity and monitoring athlete development; its reliability depends on electrode placement, signal quality, and accurate identification of muscle activation periods. This study proposes an intelligent EMG sensing framework integrating preliminary electrode placement assessment, muscle activity detection, feature extraction, and regression-based progression prediction. A placement assessment indicated that positioning the electrode adjacent to the innervation zone produced the highest RMS under the tested conditions. A two-stage activity detection method based on clustering and probabilistic modeling achieved an average error of 1.5% and a temporal deviation of 19 ms. Nine time-domain EMG features extracted from the detected activity segments were used to characterize athlete progression and estimate the time required to reach a reference neuromuscular profile. Among the methods, Linear Regression provided the best fit to the data, obtaining R^2^ = 0.987 and RMSE = 4.21 and suggesting a predominantly linear relationship between the EMG-derived features and training duration within the dataset. However, these results were obtained from only six longitudinal observation periods for a single representative athlete, with each period represented by a 90-dimensional EMG feature vector derived from the ten movement classes. Therefore, the results should be interpreted as preliminary, athlete-specific goodness-of-fit findings rather than evidence of generalizable predictive performance. Validation using larger longitudinal cohorts and independent datasets is required. The proposed framework is compatible with future IoT-enabled wearable and edge-computing architectures; however, hardware-level implementation was beyond the scope of this study.

## 1. Introduction

Human movement results from coordinated interaction between the central nervous system and the musculoskeletal system. During voluntary movement, neural commands activate motor units, generating muscle contraction and mechanical force. Surface electromyography (sEMG) provides a non-invasive means of recording this neuromuscular activity and has therefore become an important sensing modality in sports biomechanics, rehabilitation, movement analysis, and athlete monitoring [1]. EMG-derived information can support the assessment of muscle activation, coordination, fatigue, and functional performance. However, the reliability of these measurements depends strongly on electrode placement, signal quality, preprocessing, and the accurate identification of muscle activity periods [2].

Electrode positions relative to the innervation zone and tendon region can substantially affect EMG amplitude and information content. Placement directly over the innervation zone may cause phase cancellation because motor unit action potentials propagate in opposite directions, whereas placement near the tendon region generally produces weaker signals. Nevertheless, electrode placement recommendations may vary across muscles, participants, and contraction conditions. Therefore, placement assessment can support protocol development, but results obtained from a single muscle or participant should not be interpreted as universally applicable.

Accurate identification of muscle activity onset and offset is another important stage of EMG analysis [3]. Single- and dual-threshold methods are computationally simple and suitable for rapid implementation, but their performance is sensitive to noise and threshold selection [4]. Statistical approaches such as the cumulative sum (CUSUM) method and the generalized likelihood ratio test (GLRT) can provide more robust detection but require appropriate statistical assumptions and parameter settings [5]. Signal-decomposition and energy-based approaches, including singular spectrum analysis and the Teager–Kaiser energy operator, can improve sensitivity to activation changes, although they may increase computational complexity or remain sensitive to signal conditions. These limitations indicate the need for an activity detection method that combines robustness under noisy conditions with relatively low computational complexity.

Previous studies have demonstrated the potential of EMG features and machine-learning methods for sports performance analysis. Wang et al. [6] proposed a KAN neural-network model for predicting punching force in Wushu Sanda athletes following neuromuscular electrical stimulation. The model used RMS, integrated EMG, and frequency-domain features and achieved an R^2^ of 0.59. Stetter et al. [7] used artificial neural networks and wearable inertial sensors to estimate knee-joint forces during sports movements, reporting correlation coefficients ranging from 0.60 to 0.94. Li et al. [8] applied multiple-kernel relevance vector regression to EMG-based knee-angle prediction and obtained an R^2^ of 0.89. These studies demonstrate the value of wearable sensing and regression methods, but they primarily address movement mechanics or force estimation rather than longitudinal neuromuscular progression.

EMG signals have also been used to model muscle activity, fatigue, and movement trajectories. Daniel et al. [9] reviewed EMG-driven approaches for modeling muscle activity and fatigue processes. Didi and Reguig [10] compared classification- and regression-based formulations for modeling local muscle fatigue phases from EMG signals and reported that the selected modeling formulation substantially influenced the estimated fatigue dynamics. Nair et al. [11] used reassigned Morlet scalograms to estimate muscle fiber-type proportions from athletes’ EMG signals. Wang et al. [12] applied a backpropagation neural network and AdaBoost Regression to predict knee motion, achieving a reported accuracy of 93.5%. Wang et al. [13] combined EMG and inertial data with Support Vector Regression to estimate shoulder-joint motion and reported an R^2^ of 0.8059. Zhang et al. [14] used combined EMG and inertial-sensor data for regression-based estimation of muscle spasticity, with Support Vector Regression providing the best performance. Although these approaches can represent complex biomechanical relationships, their performance depends on the selected features, model parameters, sensing conditions, and available sample size.

Machine-learning methods have further been investigated for injury-risk and training-outcome prediction. Kim et al. [15] compared Ridge Regression, Random Forest, and XGBoost using RMS, MAV, IEMG, SSI, VAR, WL, ZC, and SSC features to estimate injury risk during single-leg landing in taekwondo athletes. Ridge Regression achieved an R^2^ of 0.99, demonstrating that a relatively simple and interpretable model may perform effectively when the underlying relationship is sufficiently regular. Prończuk et al. [16] used Random Forest to predict the effectiveness of EMG-guided training in badminton athletes and obtained an AUC of 0.92 and an F1-score of 0.89. These studies show the practical potential of EMG-based machine learning, although they focus on injury risk or training effectiveness rather than estimating the time needed to reach an athlete-specific reference profile.

Deep-learning methods have also been employed in continuous EMG regression. Ameri et al. [17] proposed a convolutional regression model based on high-density EMG data, while Zhang et al. [18] used a bidirectional long short-term memory model for continuous knee-joint trajectory prediction. Such models can learn complex nonlinear and temporal relationships but generally require comparatively large datasets, greater computational resources, and more extensive parameter optimization. In contrast, linear regression provides greater interpretability and computational efficiency, whereas XGBoost and CatBoost can capture nonlinear feature interactions. Support Vector Regression is effective in high-dimensional spaces but is sensitive to kernel and hyperparameter selection, while Gaussian Process Regression can quantify uncertainty but may produce excessively smooth predictions under limited-data conditions.

Overall, previous investigations have established the usefulness of EMG-derived features for movement classification, joint-motion estimation, force prediction, fatigue assessment, injury-risk evaluation, and trajectory prediction. However, most studies address only one stage of the analytical process. The integration of electrode placement assessment, robust muscle activity detection, longitudinal feature monitoring, reference profile construction, and regression-based estimation of the time required to reach a reference neuromuscular state has received limited attention. Moreover, the advantages of complex nonlinear models over simpler and more interpretable regression methods remain uncertain when longitudinal EMG datasets contain only a small number of observation periods.

The objective of the present study is to develop an intelligent EMG sensing framework for monitoring athlete development and estimating the time required to reach a reference neuromuscular profile. The underlying dataset contains EMG recordings from 30 professional national-wrestling athletes across eight weight categories and ten movement classes. The longitudinal regression analysis is presented as a subject-specific case involving a representative athlete from the −73 kg weight category, with six longitudinal observation periods collected over one month. Each observation period was represented by a 90-dimensional EMG feature vector derived from nine selected features across the ten movement classes. Consequently, the regression results are interpreted as preliminary within-subject findings rather than evidence of population-level predictive generalization.

The adopted methodology consists of a preliminary electrode placement assessment, EMG acquisition and preprocessing, two-stage muscle activity detection, feature extraction, construction of weight- and movement-specific reference profiles, and regression-based progression prediction. Nine time-domain EMG features, MAV, WL, RMS, VAR, DASDV, SSI, 4POW, AAC, and IEMG, were used as sensing biomarkers. Linear Regression, XGBoost, Support Vector Regression, CatBoost, and Gaussian Process Regression were evaluated using a nested cross-validation procedure to prevent information leakage during hyperparameter selection.

This study extends our previous work [19], which used the same underlying EMG dataset to evaluate 36 signal features, identify nine informative features, and classify ten movement classes using machine learning algorithms. The previous study focused exclusively on feature engineering and movement classification and did not include longitudinal athlete monitoring, reference profile construction, or regression-based time prediction. In contrast, the present study uses the previously identified features as sensing biomarkers and introduces reference EMG profiles for each weight category and movement class, longitudinal progression assessment, and regression-based estimation of the time required to reach a reference neuromuscular profile. Thus, the dataset and selected feature subset are reused, whereas the research objective, analytical framework, prediction target, and regression results are new.

The principal contribution of this study is the integration of these analytical stages into a unified athlete-monitoring framework. The proposed approach combines a preliminary assessment of electrode placement, probabilistic muscle activity detection, previously validated EMG features, weight- and movement-specific reference profiles, and regression-based estimation of athlete progression. The framework is also conceptually compatible with future IoT-enabled wearable and edge-computing architectures; however, embedded implementation, latency, memory usage, communication performance, and power consumption were not evaluated in the present study. The proposed workflow and its methodological stages are presented in Section 2.

## 2. Methodology

**Figure 1:**
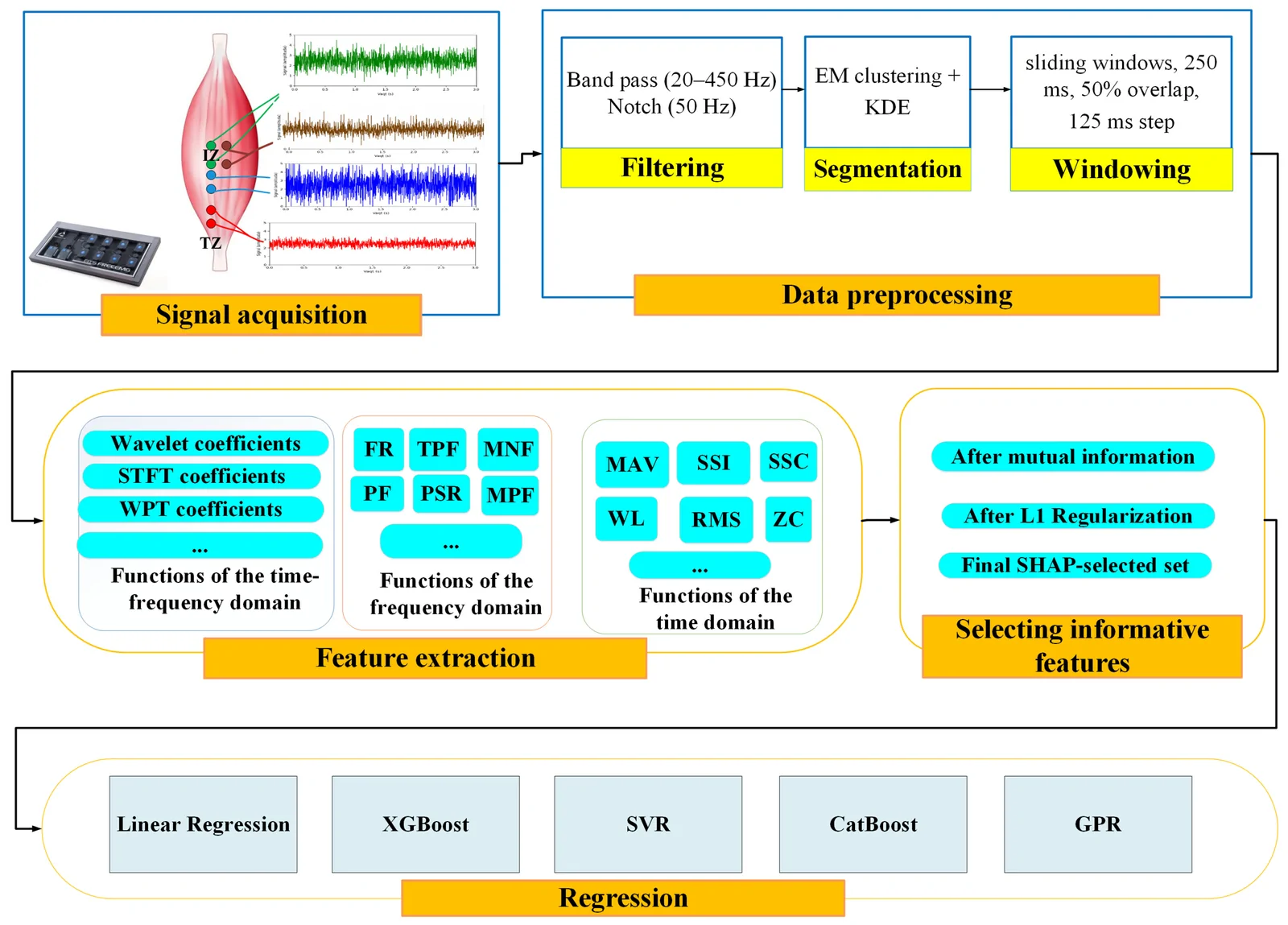
General workflow of the proposed EMG sensing framework.

The overall methodological workflow of the proposed framework is presented in Figure 1. The framework integrates EMG data acquisition and preprocessing, electrode placement assessment, muscle activity detection, feature extraction, reference profile construction, and regression-based time prediction within a unified analytical pipeline.

### 2.1. EMG Sensing Protocol for Reference Profile Prediction

**Figure 2:**
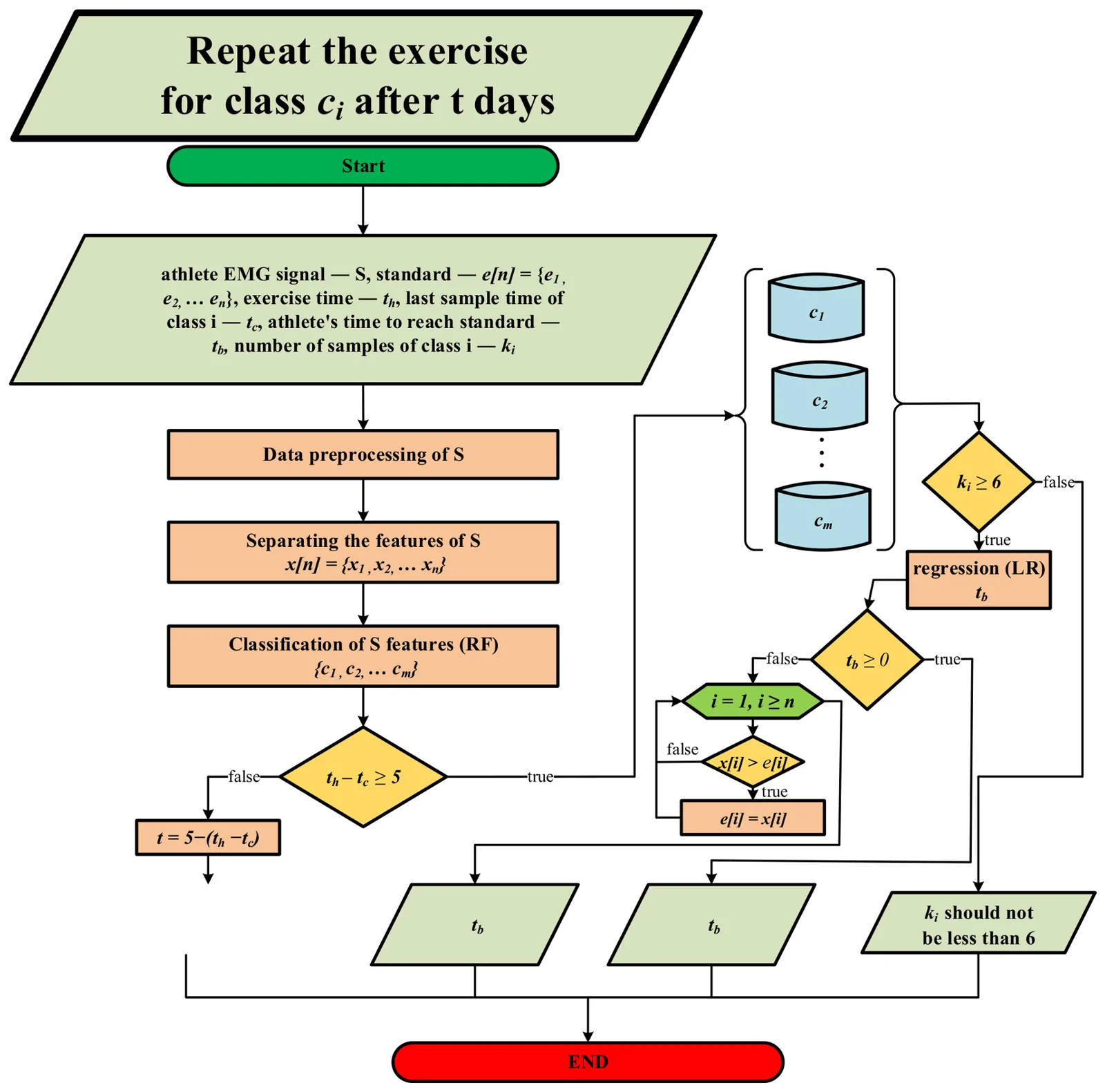
Algorithm for estimating the time to reach the reference EMG profile of an athlete.

In this study, an EMG sensing-based experimental protocol was developed to estimate and predict the time required for an athlete to reach a reference neuromuscular profile. Previous biomechanical studies on wrestlers have shown that quantitative kinematic and kinetic indicators are useful for evaluating overall fitness and movement performance; therefore, the present study extends this athlete assessment perspective by focusing on EMG-derived neuromuscular indicators [20,21]. The proposed protocol integrates EMG sensing acquisition, feature extraction, reference profile construction, similarity assessment, and regression-based prediction within a unified analytical workflow. Based on this protocol, an algorithm was developed for athlete development monitoring and reference profile time estimation using EMG sensing data (Figure 2).

This study used the EMG dataset developed in our previous work [6]. This dataset was collected from 30 professional national wrestling athletes using an 8-channel BTS FreeEMG 1000 device (BTS S.p.A., Garbagnate Milanese, Italy) and included 10 motion classes and a total of 4500 observations. In the previous study, EMG sensing data were subjected to preliminary preprocessing steps, time domain features were extracted from motion segments, and their classification efficiency was analyzed. As a result, nine highly informative EMG features (MAV, WL, RMS, VAR, DASDV, SSI, 4POW, AAC, and IEMG) were identified in our previous study [19]. The relevance of optimized informative feature subsets has also been supported by hybrid feature construction approaches, which aim to improve the compactness, discriminative ability, and computational efficiency of machine learning models [22]. These features demonstrated stable discriminative capability across multiple movement classes and machine learning algorithms. Therefore, they were selected as the initial sensing biomarkers for the proposed athlete monitoring framework.

The general characteristics of the dataset used for the regression model are summarized in Table 1.

This dataset contains nine time-domain features extracted from EMG signals, and this set of features was used in this work. The selected features were used as the main input parameters of the developed algorithm. At the prediction-preparation stage, the 30-athlete dataset was first divided by weight category, and the highest-performing athletes within each category were then selected to construct the reference EMG profiles. The weight categories used in national wrestling competitions—–60 kg, −66 kg, −73 kg, −81 kg, −90 kg, −100 kg, −120 kg and +120 kg were taken as the basis. After that, the values of nine features extracted from EMG signals for each weight category and movement class (MAV, WL, RMS, VAR, DASDV, SSI, 4POW, AAC and IEMG) were analyzed, and the maximum values observed in this group were taken as reference indicators. As a result, a separate reference EMG profile was formed for each weight category and movement class. In the next steps, the EMG data of the athlete being analyzed were compared with a reference profile corresponding only to the athlete’s weight category and the class of movement being performed. To determine which movement class the EMG signal of the athlete being analyzed belongs to, a Random Forest classifier developed in a previous study [6] was used. The classifier divides the signal into one of the corresponding movement classes (c1, c2, …, c10) based on nine features extracted from the EMG signal. The reference EMG profile corresponding to the identified class is selected, and the subsequent steps of proximity assessment and regression are performed for this class.

In this study, a regression model was used to study the relationship between the athlete’s EMG characteristics and the duration of training. The model was trained using the features extracted from the EMG signals for each observation point as input parameters, and the time (in days) since the start of training as the target variable.

After training the model, the EMG feature vector formed based on the reference group of athletes was entered into the regression model. Since the reference EMG characteristics represent the reference neuromuscular state of highly skilled athletes, the time value returned by the model was interpreted as the estimated time required for the athlete to reach this reference EMG profile.

Before the start of the experiment, the athletes were required to refrain from performing any physical exercises, not to strain the muscles, and to be in a state of sufficient rest. These requirements were aimed at minimizing the effects of muscle fatigue and ensuring the stability and reproducibility of the recorded EMG signals.

According to the protocol, the athlete under analysis provided EMG signals for a month. The athlete performed two movement classes per day, and it took five days to complete a total of 10 classes, after which this process was repeated in the same sequence on the following days. As a result, at the end of a month of observation, six independent datasets were formed for each class, which allows for a stable analysis of changes in muscle activity over time. This time-based tracking mechanism serves to assess the dynamics of the athlete’s development in the developed algorithm.

Practical experiments have shown that during some exercises, the quality of the recorded EMG signals may be insufficient due to electrode displacement, uneven muscle tension, or external noise. Therefore, on each training day, the athlete performed five exercises in the selected class. From the five recorded signal samples for each class, one signal sample was selected that was closest to the reference values. This selection process serves to generate stable and reliable data for subsequent forecasting stages. This procedure also increases sensing reliability by reducing the influence of electrode displacement, transient artifacts, and day-to-day signal variability commonly observed in wearable EMG systems.

To assess the proximity to the reference values, the criteria of cosine similarity, Euclidean distance, and correlation coefficient were calculated among the selected EMG feature vectors.

Cosine similarity assesses the degree of similarity in direction between the current and reference EMG feature vectors and is used to determine their shape matching [23].

(1) $$S_{\cos}=\frac{\sum_{i=1}^{n}x_{i}e_{i}}{\sqrt{\sum_{i=1}^{n}x_{i}^{2}}\sqrt{\sum_{i=1}^{n}e_{i}^{2}}},\;\;\;\;P_{\cos}=\frac{S_{\cos}+1}{2}\times 100\%$$

Let *x* = [*x*_1_, *x*_2_, …, *x_n_*] denote the EMG feature vector of the athlete under analysis and *e* = [*e*_1_, *e*_2_, …, *e_n_*] the corresponding reference feature vector for the same weight category and movement class. Here, *n* = 9 is the number of selected EMG features, and *x_i_* and *e_i_* represent the values of the *i*-th feature in the analyzed and reference vectors, respectively. These values correspond to MAV, WL, RMS, VAR, DASDV, SSI, 4POW, AAC, and IEMG extracted from the filtered, MVC-normalized, and segmented sEMG recordings.

In Equations (1)–(3), *S*_cos_ denotes cosine similarity, $D_{\mathrm{Euc}}$—denotes Euclidean distance, and Scorr denotes the Pearson correlation coefficient. $D_{\mathrm{Euc}}^{\max}$—is the maximum Euclidean distance observed among the candidate recordings evaluated against the same reference profile. Furthermore, $\bar{x}$ and $\bar{e}$ are the arithmetic means of the analyzed and reference feature vectors, respectively. The corresponding *P*_cos_, *P*_Euc_, and *P*_corr_ values express the three measures on a common 0–100% similarity scale.

The Euclidean distance estimates the absolute deviation of the current EMG feature values from the reference values and indicates the proximity in terms of amplitude [24].

(2) $$D_{\mathrm{Euc}}=\sqrt{\sum_{i=1}^{n}{\left(x_{i}-e_{i}\right)}^{2}},\;\;\;\;P_{\mathrm{Euc}}=\left(1-\frac{D_{\mathrm{Euc}}}{D_{\mathrm{Euc}}^{\max}}\right)\times 100\%$$

The correlation coefficient determines the degree of linear relationship between the current and reference EMG characteristics and assesses their change trend [25].

(3) $$S_{\mathrm{corr}}=\frac{\sum_{i=1}^{n}\left(x_{i}-\bar{x})(e_{i}-\bar{e}\right)}{\sqrt{\sum_{i=1}^{n}{\left(x_{i}-\bar{x}\right)}^{2}}\sqrt{\sum_{i=1}^{n}{\left(e_{i}-\bar{e}\right)}^{2}}},\;\;\;\;P_{\mathrm{corr}}=\frac{S_{\mathrm{corr}}+1}{2}\times 100\%$$

Cosine similarity, Euclidean proximity, and correlation describe complementary characteristics of the EMG feature vectors: directional agreement, absolute magnitude proximity, and similarity of variation patterns, respectively. Because all three measures were converted to the same dimensionless 0–100% scale, they were combined using an equal-weight arithmetic mean. Equal weighting was selected to avoid assigning an unsupported preference to any individual similarity measure. The resulting integral closeness indicator is defined in Equation (4):

(4) $$P_{\mathrm{int}}=\frac{P_{\cos}+P_{\mathrm{Euc}}+P_{\mathrm{corr}}}{3}$$

Here, *P*_int_ denotes the integral closeness percentage. A higher value indicates greater overall agreement between the athlete’s current EMG feature vector and the corresponding reference profile.

Because EMG signals are stochastic, feature values vary between repetitions of the same movement, and small electrode displacements, differences in muscle tension, or external noise may further degrade individual recordings. To reduce this variability, the five signals recorded for each movement class on a given training day were compared with the corresponding reference EMG profile, and the recording with the highest integral proximity value was retained as the representative sample for that class. The remaining recordings were excluded from the dataset. The integral proximity index therefore served only as a sample-selection criterion; it was not used to define the regression target variable or to fit the model parameters. This selection procedure ensured that only the most stable recordings were passed to the regression stage and limited the influence of random signal variation.

This approach allows only the closest and most stable EMG samples to the reference profile to be passed to the regression stage and reduces the influence of random variations in the signal. Thus, the proposed EMG sensing-based prediction protocol enables step-by-step monitoring of athlete development and provides a reliable estimation of the time required to reach a reference neuromuscular profile. By integrating data acquisition, feature-level analysis, similarity assessment, and predictive modeling, the protocol supports intelligent athlete monitoring and long-term performance assessment. The proposed workflow can be viewed as a complete intelligent EMG sensing pipeline that transforms raw biosignals into clinically and practically meaningful indicators of athlete development.

**Figure 3:**
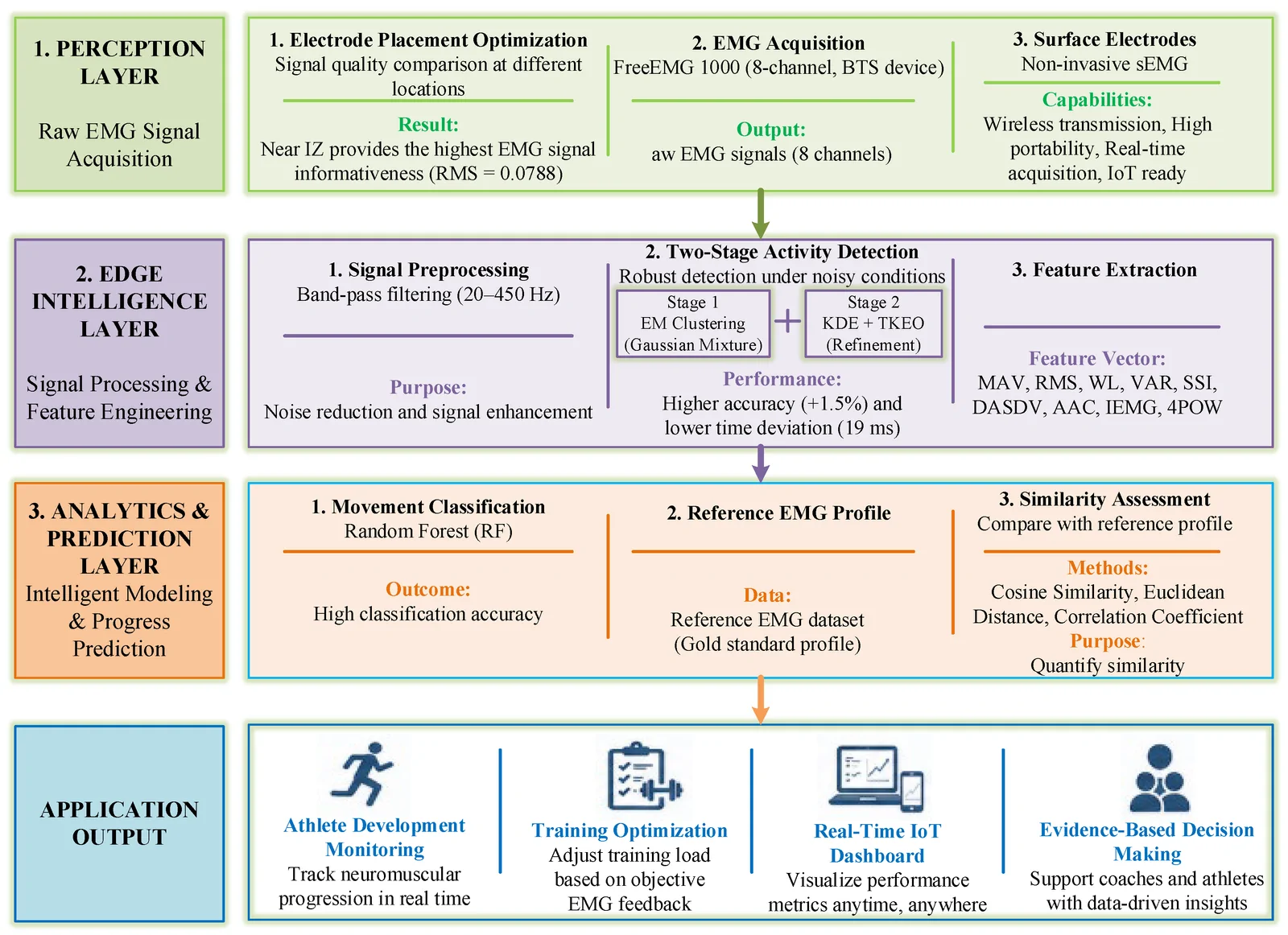
Conceptual three-tier IoT architecture for EMG-based athlete monitoring.

From a conceptual IoT perspective, the proposed sensing pipeline can be mapped onto a three-tier wearable architecture. The perception layer includes EMG acquisition, the edge/transport layer includes preprocessing and feature extraction, and the application layer includes regression-based prediction. Figure 3 presents a conceptual mapping rather than a validated hardware implementation. Latency, memory usage, communication performance, and power consumption were not evaluated in this study.

### 2.2. EMG Sensing Data Acquisition and Preprocessing

**Figure 4:**
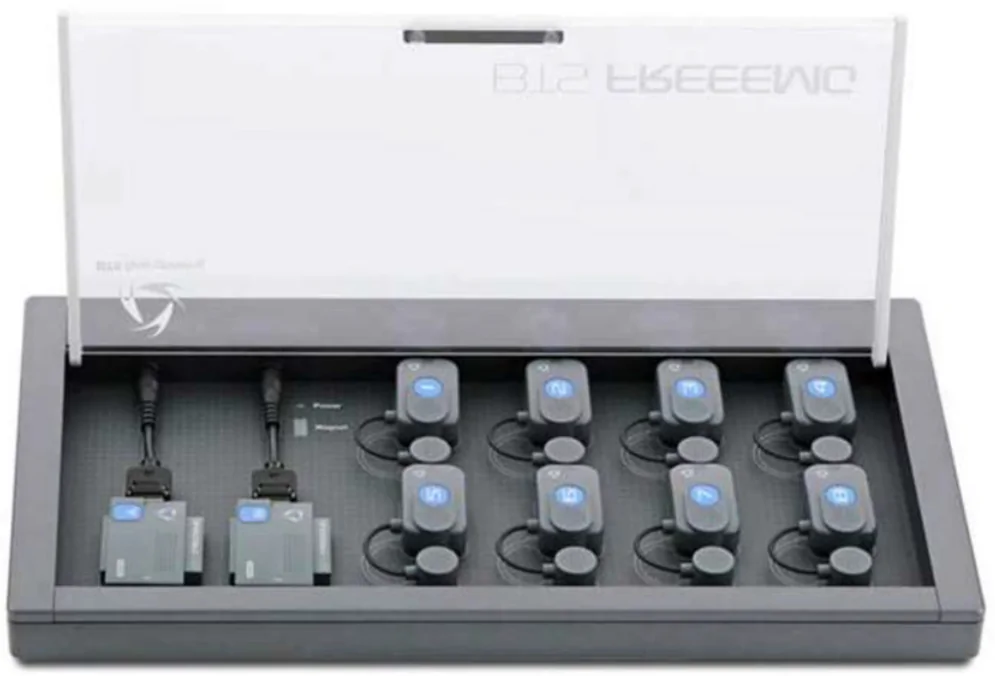
General view of the FreeEMG electromyography device.

In this study, an eight-channel FREEEMG 1000 electromyography device (BTS S.p.A., Garbagnate Milanese, Italy) was used to record the athletes’ muscle activity (Figure 4).

This device was used to acquire EMG sensing data from multiple muscle groups simultaneously with high accuracy. The muscle groups recorded in the study and their corresponding EMG channels are listed in Table 2.

The FreeEMG system is based on non-invasive surface electrodes that are placed on the muscle surface and record the signal directly. During the experiment, EMG sensing data were monitored in real time and stored for subsequent sensing analysis. The high accuracy of this device allows for reliable assessment of muscle activity, tension, and functional status during contraction [26].

Following data acquisition, all EMG recordings were subjected to the same preprocessing workflow before muscle activity detection and feature extraction.

Since EMG sensing data are highly susceptible to noise, a multi-stage processing process was performed to prepare them for further analysis. Initially, a band-pass filter was used to reduce low- and high-frequency noise in the signal. The 20–450 Hz range was selected to preserve useful EMG components during the filtering process. In addition, noise components at a frequency of 50 Hz were suppressed to reduce the effect of the power grid.

Following filtering, amplitude normalization was performed using a maximum voluntary contraction (MVC) reference recorded separately for each participant and muscle under standardized conditions. The amplitude of each EMG recording was expressed relative to its corresponding MVC value before signal segmentation and feature extraction. The same subject- and muscle-specific normalization procedure was applied consistently throughout the recording sessions to reduce variability caused by differences in signal amplitude and to improve inter-subject and inter-session comparability.

After the filtering stage, the EMG signal was divided into time segments. Consecutive time windows of 200 ms (0% overlap) were used during the segmentation process. This approach allows analyzing the signal in local time intervals, identifying dynamic changes in muscle activity, and calculating features stably and reliably.

These preprocessing stages served to improve the quality of EMG sensing data, extract informative components of the signal, and increase the accuracy of feature detection and activity periods in subsequent stages. Reliable preprocessing is a fundamental requirement for intelligent sensing applications because prediction accuracy strongly depends on the quality of the acquired physiological signals.

These preprocessing steps produced consistently filtered, normalized, and segmented EMG data for the subsequent activity detection and feature extraction stages.

### 2.3. Preliminary Electrode Placement Assessment

This single-subject, single-channel experiment was conducted as a preliminary pilot assessment to determine a practical electrode placement criterion before the main data-acquisition stage. It was not intended as a population-level validation across all subjects and muscle groups.

**Figure 5:**
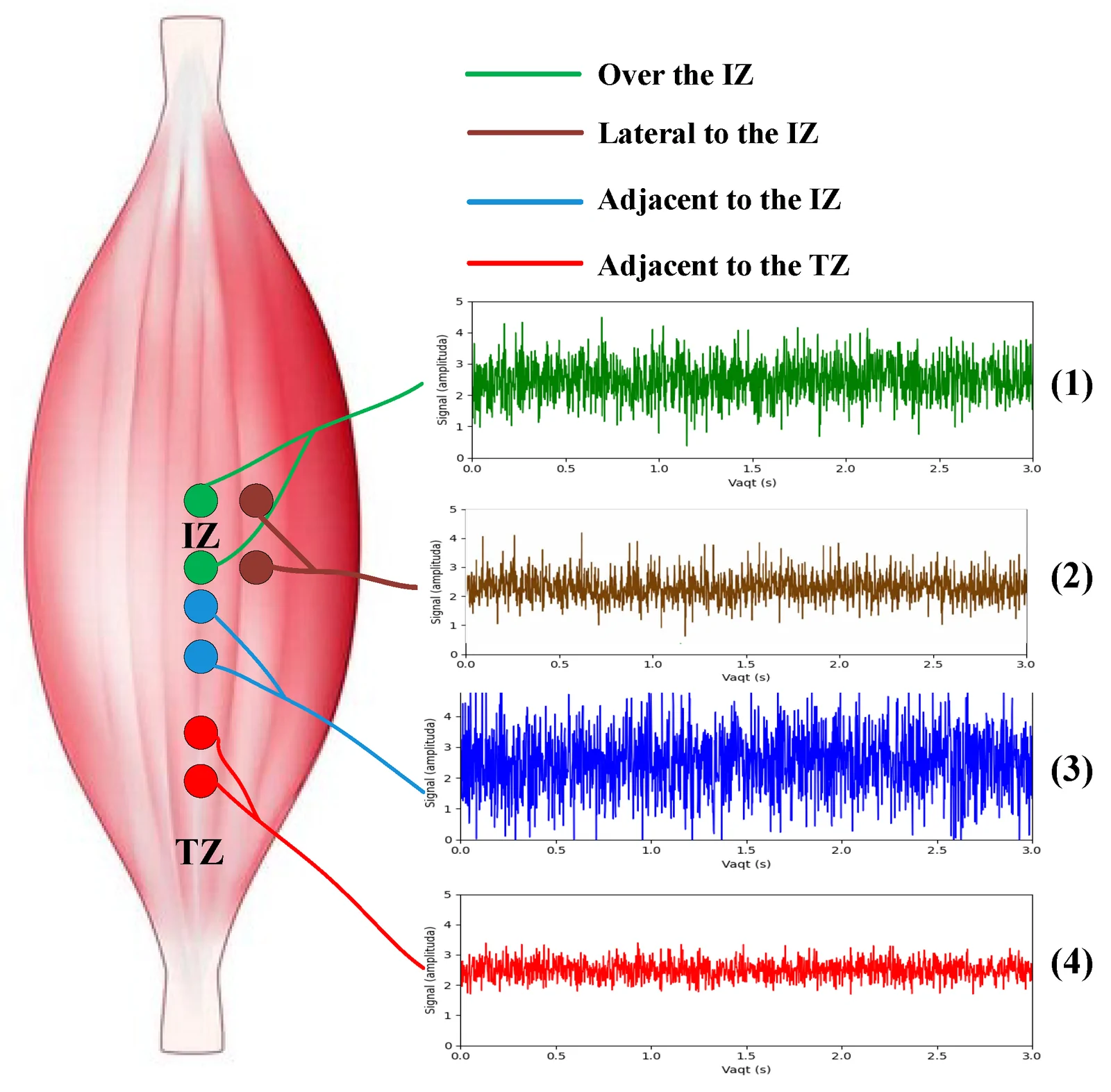
Variation in EMG amplitude depending on electrode location: (1) over the innervation zone (IZ); (2) lateral to the IZ; (3) adjacent to the IZ; and (4) adjacent to the tendon zone (TZ).

A single channel of the FreeEMG device was used to perform this procedure. During the experiment, a single subject performed a standing fist exercise. The electrodes were placed at four different locations along the muscle, defined as over the IZ, lateral to the IZ, adjacent to the IZ, and adjacent to the TZ. In this study, over the IZ denotes placement directly above the innervation zone, lateral to the IZ denotes placement to the side of the innervation zone, adjacent to the IZ denotes placement next to the innervation zone within the muscle belly, and adjacent to the TZ denotes placement next to, but not directly over, the tendon zone. These four terms are used consistently throughout the manuscript. In each case, the amplitude of the recorded EMG signals was visually analyzed, and the effect of sensor placement on EMG sensing quality was assessed (Figure 5).

The results presented in Figure 5 show that EMG amplitude varied depending on electrode position along the muscle. The signal recorded adjacent to the innervation zone (IZ), the region containing neuromuscular junctions, exhibited the highest amplitude, likely reflecting strong motor-unit activity in this region. In contrast, placement over the IZ produced a lower amplitude because motor unit action potentials (MUAPs) propagate in opposite directions from this region, potentially causing phase cancellation. Signals recorded lateral to the IZ also showed relatively lower amplitudes because of the increased distance from the principal signal sources.

The lowest EMG amplitude was observed adjacent to the tendon zone (TZ), where the muscle fibers approach and connect to the tendon tissue and detectable neuromuscular activity is reduced. Although visual inspection indicated differences among the electrode positions, a quantitative comparison was required. Therefore, the root mean square (RMS), which quantifies the effective amplitude of the EMG signal over a specified time window, was calculated for each electrode position using Equation (5) [6].

(5) $$RMS=\sqrt{\frac{1}{N}\sum_{i=0}^{N}{\left|x_{i}\right|}^{2}}$$

RMS is calculated as the square root of the mean of the squared signal amplitudes and provides a quantitative measure of the effective EMG amplitude over a specified time window. On this basis, the RMS values of the signals recorded over the IZ, lateral to the IZ, adjacent to the IZ, and adjacent to the TZ were compared to quantitatively evaluate the four electrode placement positions (Table 3).

As shown by the RMS values presented in Table 3, the effective amplitude of the recorded EMG signals varied across the four electrode locations. The highest RMS value was obtained adjacent to the IZ (0.0788), indicating a stronger recorded EMG signal under this placement condition. This may be attributed to the concentration of neuromuscular junctions around the IZ and the resulting motor-unit activity. The RMS values recorded over the IZ (0.0707) and lateral to the IZ (0.0669) were lower, potentially because of MUAP phase cancellation and the increased distance from the principal signal sources. In particular, the bidirectional propagation of MUAPs from the IZ may cause partial phase cancellation and reduce the recorded signal amplitude. The lowest RMS value (0.0523) was obtained adjacent to the TZ, where the muscle fibers approach the tendon tissue and detectable neuromuscular activity is comparatively limited.

Under the tested conditions, the position adjacent to the IZ produced the highest RMS value among the four evaluated locations. This pilot result was used only to provide practical guidance for the subsequent electrode placement procedure and should not be interpreted as evidence that this position is universally optimal across all muscles and athletes.

### 2.4. Algorithm for Detecting Muscle Activity Segments in EMG Signals

Various algorithmic approaches have been developed to detect the onset and termination of muscle activity in EMG signals. The simplest methods include single-threshold methods based on monitoring the signal amplitude exceeding a certain threshold value [27]. Although these approaches are computationally simple, they are sensitive to noise and the accuracy depends on the selected parameters.

Double-threshold methods and statistical approaches, including CUSUM and other probabilistic models, have been proposed to increase accuracy. Signal decomposition methods, such as singular spectral analysis (SSA), are also effective in reducing noise and extracting useful components. More recently, learning-based approaches have also been applied to the automatic delineation of structures and events in biomedical signals and images, where data-driven models are used instead of manually tuned decision rules [28]. However, most of these approaches are parameter-dependent and have limitations in stable operation in real noisy EMG signals.

The process of detecting muscle activity from EMG sensing data is complicated by background noise, artifacts, and gradual changes in signal amplitude. Therefore, traditional methods often fail to accurately and stably detect activity thresholds. Therefore, this study proposes a two-stage EMG activity detection method for identifying muscle activation periods from EMG sensing data.

In the proposed method, EMG onset detection is performed in two sequential stages. In the first stage, the signal is analyzed in its entirety and the region where the activity is likely to occur is roughly determined. In the second stage, the search is limited to this region and the goal is to find the onset time of EMG activity with high accuracy. The complete procedure is summarized in Algorithm 1.
**Algorithm 1.** Detecting the onset of muscle activity in the EMG signal**Input:** x—EMG signal, N—signal length/* Step 1: Determine the area of activity */1  Peak ← local maxima detection(x)2  Cluster ← Separation into 2 groups using EM      - muscle activity      - background noise3  PDF_activity_ ← Kernel Density Estimation (KDE) for muscle activity4  PDF_noise_ ← KDE for background noise5   **for** n ← 1 **to** N **do**6       **if** PDF_activity_[n] **>** PDF_noise_[n] **then**7           active_region_ ← n8       **end**9   **end**10  onset_rough_ ← active_region_ starting point***/ Stage 2: Onset clarification /***11  W ← signal window around onset_rough_12  Energy ← Teager–Kaiser energy operator13  onset_final_ ← the point where energy changes the most14  return onset_final_**Output:** onset_final_—starting point of muscle activity

Given the absolute filtered and normalized EMG signal *x*[*n*], *n* = 1, …, *N*, the algorithm first identifies the local maxima *x*[*l_i_*], where *l_i_* denotes the sample location of the *i*-th peak. A two-component Expectation–Maximization model assigns each detected peak a label *d_i_*, where *d_i_* = 1 represents muscle activity and *d_i_* = 0 represents background noise. Gaussian kernel density estimation with bandwidth is then applied to the temporal locations of both groups, producing the activity and noise probability density functions PDF_activity_ and PDF_noise_. Their intersection points define candidate activity intervals, and the largest interval provides the preliminary onset and offset estimates. Finally, these estimates are refined within a local window *W* using the Teager–Kaiser energy operator, and the samples exhibiting the greatest energy changes are selected as the final activity boundaries.

**Figure 6:**
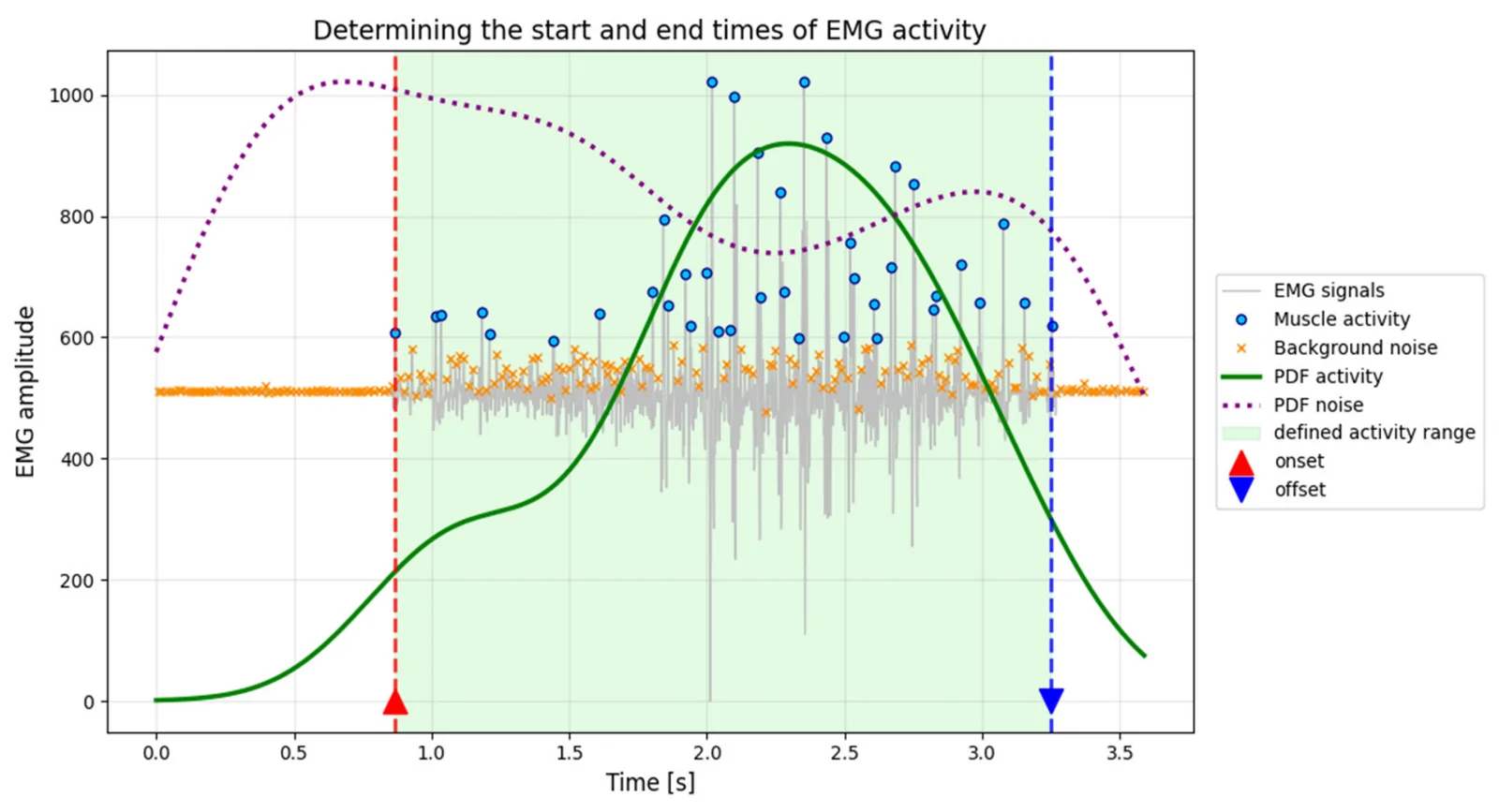
Detection of the onset and termination of EMG activity. The red dashed vertical line with the upward-pointing triangular marker indicates the onset of muscle activity, whereas the blue dashed vertical line with the downward-pointing triangular marker indicates its termination.

Here, *N* is the total number of EMG samples, *l_i_* is the location of the *i*-th local maximum, *d_i_* is its EM-assigned class label, *h* is the KDE bandwidth, and *W* is the local refinement window length. The parameters *h* and *W* control the smoothness of the estimated probability density functions and the temporal range of boundary refinement, respectively. The peak-detection threshold separates relevant local maxima from minor signal fluctuations, whereas the EM convergence tolerance determines when the iterative clustering procedure terminates. Identical parameter-selection criteria were applied to all evaluated signals. The operating principle of the proposed two-stage activity detection algorithm is illustrated in Figure 6.

In this figure, the gray signal represents the absolute value of the EMG signal. The blue circles indicate local maxima related to muscle activity, and the yellow crosses indicate local maxima related to background noise. The green solid line represents the PDF corresponding to muscle activity pulses, and the purple dotted line represents the PDF corresponding to background noise pulses. These PDFs were generated using the KDE method to smoothly represent the time distribution of local maxima. The light green area represents the active region of the EMG signal. The red vertical line and the upward marker indicate the start point of muscle activity, and the blue vertical line and the downward marker indicate the end point of muscle activity. Accurate identification of activation periods is particularly important for intelligent wearable sensing systems because noisy conditions, motion artifacts, and signal variability may significantly affect the reliability of extracted EMG biomarkers. Therefore, robust activity detection serves as a critical component for ensuring reliable neuromuscular monitoring and subsequent predictive analysis.

Table 4 presents a controlled comparison of the algorithms used to detect the onset of muscle activity. All methods were implemented by the authors and reevaluated using the same EMG dataset, identical preprocessing procedure, and the same manually identified activity-onset reference points. None of the numerical results were taken directly from the cited literature. The parameters of each method were selected according to their original descriptions and kept fixed throughout the evaluation. Average error and temporal deviation were calculated using identical criteria for all methods. According to the analysis results, the single-threshold method showed the highest error, with an average error of 3.1% and an average deviation of 32.0 ms. This indicates that this method is sensitive to noise and does not detect activity stably. In the dual-threshold method, the average error decreased to 2.6% and the average deviation was 23.0 ms. This indicates that the accuracy and reliability have been improved compared with the single-threshold method. In the methods based on statistical optimal decision-making (CUSUM/GLRT), the average error was 2.1% and the average deviation was 21.0 ms, and the results were relatively stable. Although the average error in the SSA-based method is at the level of 2.0%, the average deviation of 27.0 ms indicates that this method tends to detect the onset late in some cases. While the Komi algorithm showed average results with an error of 2.2% and a deviation of 24.0 ms, the TKEO-based method is characterized by relatively high accuracy with an error of 1.9% and a deviation of 21.0 ms.

The proposed method showed the best results among all considered algorithms. In this method, the average error was 1.5% and the average deviation was 19.0 ms, and the onset of activity was determined at the optimal location in the signal. The obtained results confirm that the proposed algorithm provides high accuracy and stability in determining the moments of activity of the EMG signal. Accurate identification of activation periods is particularly important for wearable sensing environments, where noisy conditions and motion artifacts may significantly affect the reliability of extracted EMG biomarkers.

### 2.5. Feature Extraction

In this study, time-domain features were extracted from EMG signals to quantitatively assess muscle activity. EMG signals contain important information reflecting muscle performance, tension level, and fatigue state.

In our previous study [6], 36 features describing EMG signals were analyzed and their effectiveness in distinguishing movements was evaluated using various classification algorithms. As a result, nine features with the highest informativeness in representing muscle activity (MAV, WL, RMS, VAR, DASDV, SSI, 4POW, AAC, and IEMG) were identified.

In this study, a three-stage feature selection process was performed to further confirm the importance of these features in the regression problem. In the first stage, 18 features with high correlation with the target variable were selected using the Mutual Information method. In the second stage, L1-regularization was applied to remove less significant and redundant features and retain the nine most important features. In the final stage, the contribution of the selected features to the model prediction was assessed using SHAP (SHapley Additive exPlanations) analysis, and the high informativeness of the nine features identified in the previous study was confirmed. The consistency of the selected features across both classification and prediction tasks confirms their robustness as sensing biomarkers for neuromuscular monitoring applications.

The overall results of the feature selection process are presented in Table 5.

The final feature subset is largely consistent with the findings reported in our previous classification study [6], where these features demonstrated high discriminative power for athlete-related EMG analysis. This consistency supports their general applicability across different intelligent sensing tasks. The nine selected features describe the dynamic changes in amplitude, energy, and time of the EMG signal. Therefore, they were used as input parameters of regression models to assess the level of neuromuscular activity of the athlete and predict the time to reach the reference EMG profile. These features and their mathematical expressions are presented in Table 6.

The nine selected features provide complementary information for the prediction task. MAV and RMS describe the overall muscle activation amplitude, whereas IEMG represents accumulated muscle activity over the analyzed segment. SSI and 4POW emphasize signal energy and high-amplitude contractions. VAR characterizes amplitude dispersion, while WL and AAC describe temporal variation and waveform complexity. DASDV measures sample-to-sample variability and is sensitive to changes in neuromuscular activation. Their combined use therefore enables the regression models to represent amplitude, energy, cumulative activity, and temporal complexity changes associated with athlete progression.

The features extracted from EMG signals are important in assessing the functional state of muscles, in particular in determining their force production capacity, endurance and fatigue level. Therefore, these features were used as input parameters for regression models in the subsequent stages.

### 2.6. Regression Models for Time Prediction

For each of the six longitudinal observation periods, nine selected EMG features were obtained from each of the ten movement classes. The resulting feature values were concatenated in a fixed movement class and feature order to form a single 90-dimensional input vector for each observation period (9 features × 10 movement classes). Thus, the regression dataset consisted of six samples (*n* = 6), each represented by 90 predictors (*p* = 90), with training time in days used as the target variable. Linear Regression, XGBoost, SVR, CatBoost, and GPR were each fitted using the complete 90-dimensional input vectors. The models were not fitted separately for individual movement classes.

Five regression models were selected a priori to represent distinct modeling approaches suitable for continuous tabular EMG data. Linear Regression was included as an interpretable and computationally efficient baseline; XGBoost and CatBoost represented nonlinear tree-based ensembles with different regularization strategies; SVR represented kernel-based modeling of complex feature relationships; and GPR represented probabilistic prediction with uncertainty estimation [29,30,31,32,33]. The purpose was to determine whether increased nonlinear model complexity provided a measurable advantage over a simple linear relationship rather than merely to evaluate a large number of algorithms. GridSearchCV was used to optimize the model hyperparameters, with the evaluated ranges presented in Table 7.

Model performance was evaluated using four complementary indicators: Mean Squared Error (MSE), Root Mean Squared Error (RMSE), Mean Absolute Percentage Error (MAPE), and the coefficient of determination (R^2^) [34]. MSE emphasizes larger prediction errors through squared deviations, whereas RMSE expresses the prediction error in the same unit as the target variable (days). MAPE represents the relative prediction error as a percentage, while R^2^ indicates the proportion of variance in the observed values explained by the model. Using these complementary metrics enables model performance to be assessed from absolute, relative, and goodness-of-fit perspectives. Lower MSE, RMSE, and MAPE values and a higher R^2^ value indicate better predictive performance.

Model Validation. To prevent information leakage during hyperparameter optimization, model evaluation was performed using nested cross-validation. In the outer loop, LOOCV was used to retain one observation as an independent test sample. Within each outer training fold, GridSearchCV was applied only to the remaining training observations to select the optimal hyperparameters. The selected model was then retrained on the complete outer training fold and evaluated on the held-out observation. This procedure was repeated until every observation had served once as the independent test sample. Thus, the test observation was not used during preprocessing, hyperparameter selection, or model training. The nested LOOCV procedure was selected because of the limited number of longitudinal observations.

## 3. Results

Because of the limited dataset size, the performance of the regression models was evaluated using the LOOCV method. This approach enabled the assessment of model stability on a relatively small set of individual observations. The complete dataset consisted of 30 athletes and 4500 observations, which were used to identify and classify ten predefined movement classes. The regression analysis was performed using the joint 90-dimensional EMG representation of all ten movement classes to estimate the time required for the athlete to reach the complete reference neuromuscular profile. Each of the six longitudinal observation periods constituted one regression sample containing 90 EMG feature values arranged in a fixed movement class and feature order. The results reported in this section correspond to a representative athlete from the −73 kg weight category, based on observation data collected over a one-month period. Accordingly, the regression analysis was designed as a subject-specific proof-of-concept comparison of different modeling assumptions rather than as population-level validation of predictive generalizability. The five regression models were evaluated using the same longitudinal dataset and therefore should not be interpreted as five independent validations of the proposed framework. The resulting subject-specific performance metrics are presented in Table 8.

Linear Regression obtained RMSE = 4.21, MAPE = 0.03, MSE = 17.72, and R^2^ = 0.987. These were the lowest RMSE, MAPE, and MSE values and the highest R^2^ value among the evaluated models. CatBoost achieved RMSE = 5.89, MAPE = 0.05, MSE = 34.69, and R^2^ = 0.978. XGBoost obtained RMSE = 6.12, MAPE = 0.05, MSE = 33.45, and R^2^ = 0.975. The corresponding results for SVR were RMSE = 7.21, MAPE = 0.06, MSE = 52.09, and R^2^ = 0.962. GPR obtained RMSE = 6.74, MAPE = 0.06, MSE = 45.43, and R^2^ = 0.932.

For two-dimensional visualization, the 90 EMG feature values obtained from nine selected features across ten movement classes were standardized and combined into a regression-weighted composite EMG score, termed the General Index (GI):

(6) $$GI_{i}=\sum_{j=1}^{90}\beta_{j}z_{i,j},$$

In Equation (6), *z_i_*_,*j*_ denotes the standardized value of the *j*-th EMG feature for observation period *i*, and *βj* denotes the corresponding coefficient learned by the Linear Regression model. The index *j* = 1,…,90 represents the nine EMG features from each of the ten movement classes arranged in a fixed movement class and feature order. The 90 coefficients were learned directly by a single Linear Regression model fitted using the complete 90-dimensional input vectors. They were not obtained by concatenating coefficients from ten separately fitted class-specific models or by fitting an additional pooled regression model.

**Figure 7:**
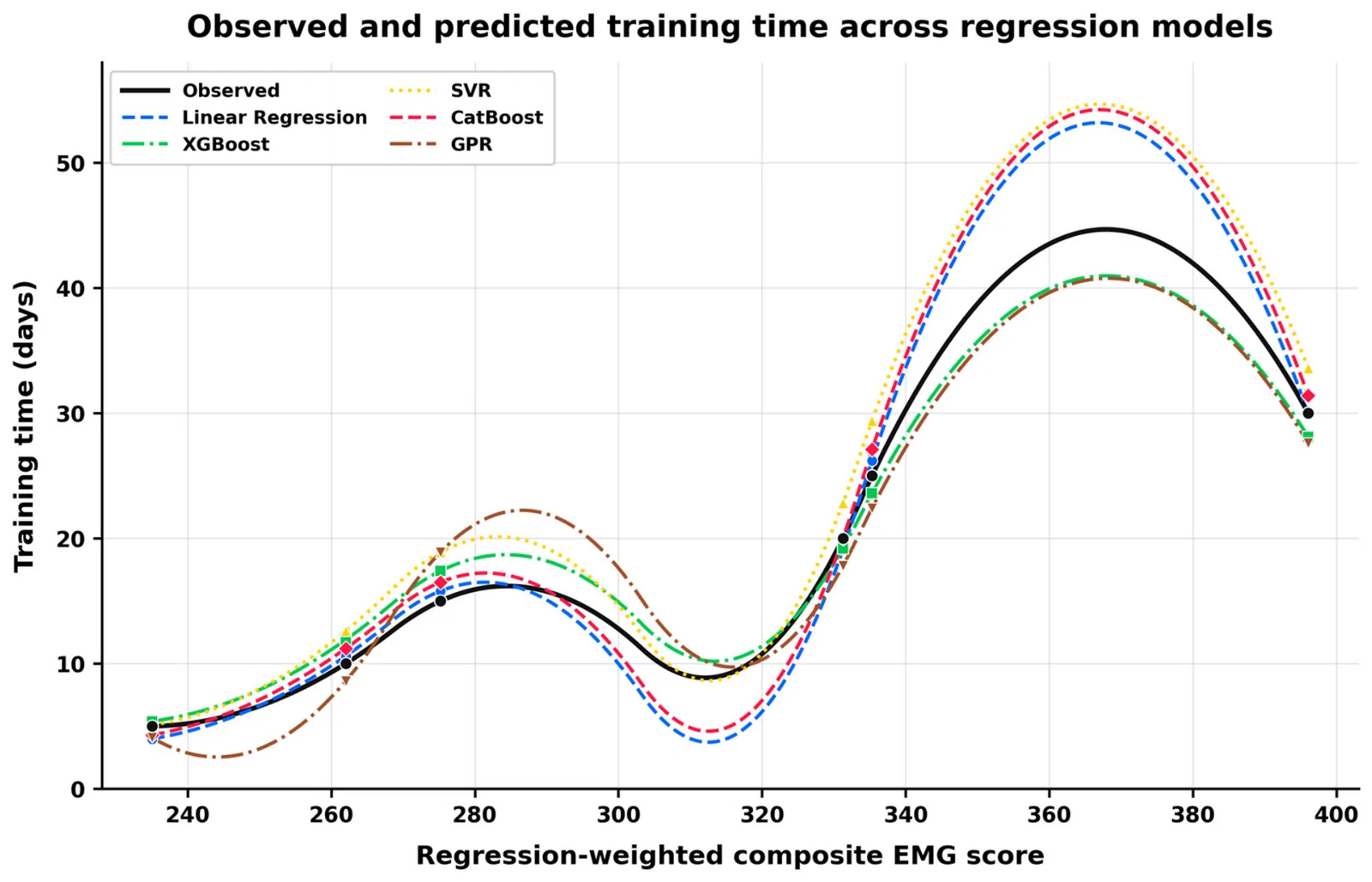
Qualitative comparison of observed and predicted training times across five regression models. The *x*-axis represents the regression-weighted composite EMG score (GI), and the *y*-axis represents training time (days). The visualization is illustrative only; model ranking is based on Table 8.

The results shown in Figure 7 correspond to six longitudinal observation periods for a representative athlete from the −73 kg weight category. The General Index was used only as a common horizontal axis for two-dimensional visualization and was not used as an input for model training, prediction, cross-validation, or performance metric calculation. Because GI is constructed using coefficients learned by Linear Regression, the shared *x*-axis is not fully model-neutral and may visually favor the Linear Regression representation. Therefore, Figure 7 should be interpreted only as a qualitative illustrative aid and not as independent confirmation of the model ranking reported in Table 8. Model performance and ranking were determined exclusively from the cross-validated RMSE, MAPE, MSE, and R^2^ values. The interpolated curves were included only for visual guidance.

Figure 7 shows that the Linear Regression prediction curve remains closest to the observed values across most of the six longitudinal observation points. Only small deviations are visible at several points, and the predicted curve follows the overall progression of the observed values. This qualitative visual pattern corresponds to the numerical results reported for Linear Regression in Table 8 (RMSE = 4.21, MAPE = 0.03, MSE = 17.72, and R^2^ = 0.987); however, Figure 7 is not used as an independent basis for model ranking.

The XGBoost prediction curve captures the overall pattern of the observed training-time values. However, greater deviations and sharper fluctuations are visible at some plotted observations. The model achieved RMSE = 6.12, MAPE = 0.05, MSE = 33.45, and R^2^ = 0.975.

The SVR prediction curve exhibits larger deviations from the observed values than the other evaluated models at several plotted observations. SVR produced RMSE = 7.21, MAPE = 0.06, MSE = 52.09, and R^2^ = 0.962. It therefore recorded the highest RMSE and MSE values among the five models.

The CatBoost prediction curve follows the general progression pattern and remains relatively stable across the analyzed observations. Nevertheless, deviations from the observed values are visible at several points. CatBoost obtained RMSE = 5.89, MAPE = 0.05, MSE = 34.69, and R^2^ = 0.978.

The GPR prediction curve represents the general trend of the observed values, although its agreement with some local variations is weaker. The model achieved RMSE = 6.74, MAPE = 0.06, MSE = 45.43, and R^2^ = 0.932. Although its RMSE was lower than that of SVR, GPR recorded the lowest R^2^ value among the evaluated models.

Overall, the cross-validated numerical metrics reported in Table 8 identify Linear Regression as the best-fitting model within the analyzed dataset. Figure 7 provides only a qualitative illustration of the observed and predicted patterns on the Linear-Regression-weighted GI axis and is not used as an independent basis for model ranking. According to the numerical metrics in Table 8, CatBoost and XGBoost provided the next-best results, whereas SVR produced the largest error values and GPR obtained the lowest coefficient of determination.

## 4. Discussion

The comparison results indicate that the relationship between the selected EMG features and training duration was predominantly linear within the analyzed dataset. Linear Regression achieved the lowest prediction errors and the highest coefficient of determination, with RMSE = 4.21, MAPE = 0.03, MSE = 17.72, and R^2^ = 0.987. Its superior performance suggests that a simple linear representation was sufficient to describe the observed athlete-specific progression pattern under the available data conditions.

The XGBoost and CatBoost models also captured the overall progression trend, but their prediction performance was lower than that of Linear Regression. XGBoost recorded RMSE = 6.12 and R^2^ = 0.975, and sharper fluctuations were observed in some regions of its prediction curve. CatBoost produced relatively stable results, with RMSE = 5.89 and R^2^ = 0.978. However, the additional complexity of these tree-based ensemble algorithms did not provide a predictive advantage over the simpler linear model. This may be because the available longitudinal observations exhibited relatively smooth and consistent temporal dynamics rather than strong nonlinear relationships.

SVR produced the highest error indicators, with RMSE = 7.21 and MSE = 52.09, despite achieving R^2^ = 0.962. The performance of SVR may have been affected by its sensitivity to kernel and hyperparameter selection under limited-data conditions. In addition, the complex and noisy characteristics of EMG signals can influence the stability of kernel-based regression when only a small number of longitudinal observations are available.

GPR obtained RMSE = 6.74, MSE = 45.43, and R^2^ = 0.932. Although its probabilistic formulation is advantageous for representing prediction uncertainty, the model did not adequately reproduce all local variations in the present data. Its comparatively low R^2^ value may be associated with the smoothing behavior of the selected Gaussian process kernel.

Overall, the advanced nonlinear models—XGBoost, CatBoost, SVR, and GPR—were able to capture the general progression trend but did not provide additional predictive benefits under the available data conditions. XGBoost and CatBoost achieved R^2^ values of 0.975 and 0.978, respectively, while SVR and GPR obtained R^2^ values of 0.962 and 0.932. These findings indicate that increased model complexity does not necessarily improve prediction when the available longitudinal data exhibit a relatively simple progression pattern.

The computational simplicity and interpretability of Linear Regression make it a potentially practical option for individualized EMG-based athlete monitoring. A simpler model enables the relationship between EMG-derived features and training duration to be interpreted more directly and requires fewer computational resources than the evaluated nonlinear alternatives. This characteristic may be beneficial for future wearable or edge-based athlete-monitoring applications.

The five-model comparison should be interpreted within the scope of the available data. Although the complete EMG dataset included 30 athletes and was used for feature identification, movement classification, and reference profile construction, the longitudinal regression analysis was based on only six longitudinal observation periods for one representative athlete, with each period represented by a 90-dimensional EMG feature vector derived from all ten movement classes. Consequently, the reported performance metrics describe comparative, athlete-specific goodness of fit and do not establish predictive generalization across athletes, weight categories, or training environments.

Within these limitations, the results suggest that EMG-derived features may provide useful physiological indicators for individualized athlete progression monitoring, training adaptation assessment, and reference profile comparison. The proposed approach may support data-driven training planning and personalized performance management, subject to broader longitudinal and external validation. Broader practical adoption of such systems will also depend on how effectively the resulting analytical outputs are communicated to coaches and athletes, since AI-based decision-support tools in health and rehabilitation contexts increasingly emphasize the transformation of complex physiological information into interpretable and actionable guidance [35].

### Research Limitations and Future Prospects

Several limitations of this study should be acknowledged. First, the reference values were derived from the maximum feature values observed within the available dataset for each movement class. Although this approach provides a practical internal reference framework, the resulting values may not represent the maximum physiological capabilities attainable by elite athletes. Therefore, the prediction results should be interpreted relative to the reference profiles established within the current dataset.

Second, the reference database was not constructed using EMG data collected from world-class or internationally ranked athletes. Consequently, the proposed framework evaluates athlete progression relative to the highest-performing individuals represented in the current dataset rather than to global elite performance standards. Future studies should incorporate athletes from broader performance levels, including world- and continental-level competitors, to establish more representative reference profiles.

Third, although the complete dataset included 30 athletes and was used for feature identification, movement classification, and reference profile construction, the longitudinal regression analysis was limited to six longitudinal observation periods for one representative athlete. Each observation period was represented by a 90-dimensional EMG feature vector derived from nine selected features across the ten movement classes. Thus, the reported regression metrics primarily describe goodness of fit to the observed athlete-specific trajectory and should not be interpreted as evidence of predictive generalization across athletes, weight categories, sports disciplines, or training environments. Larger longitudinal cohorts and independent test datasets are required to validate the present preliminary findings.

Fourth, EMG measurements are sensitive to electrode placement, skin impedance, motion artifacts, cross-talk between neighboring muscles, and external electromagnetic interference. Although standardized acquisition, amplitude normalization, preprocessing, and filtering procedures were applied, residual variability may still have influenced the extracted features and prediction performance.

Fifth, model evaluation was performed using nested cross-validation, with LOOCV in the outer loop and hyperparameter selection restricted to the corresponding training folds. This procedure reduced the risk of information leakage under limited-data conditions but cannot replace external validation. Further evaluation using independent longitudinal datasets is therefore required to assess the robustness and generalizability of the models.

Sixth, the electrode placement comparison was limited to one subject, one muscle, and one contraction task; therefore, further validation across multiple subjects, muscle groups, and contraction conditions is required.

Seventh, the proposed IoT and edge-computing architecture remains conceptual. Embedded implementation and measurements of latency, memory usage, communication performance, and power consumption were not performed and require future investigation.

Future research should focus on constructing a larger longitudinal EMG database involving athletes from different weight categories, performance levels, and training backgrounds. The inclusion of elite athletes would enable the development of more representative reference profiles, while independent cohorts would allow external evaluation of prediction performance. Further studies should also investigate additional EMG-derived features and alternative learning approaches after sufficient longitudinal data become available.

The practical implementation of the proposed framework should be evaluated using low-power wireless sensor nodes and embedded or edge-computing platforms. Such evaluation should include measurements of processing latency, memory requirements, communication reliability, and power consumption before claims regarding real-time or scalable IoT deployment can be validated. Long-term monitoring studies may subsequently support a more reliable understanding of neuromuscular adaptation and individualized athlete-development trajectories.

## 5. Conclusions

This study presented an integrated EMG-based framework for monitoring athlete development and estimating the time required to reach a reference neuromuscular profile. The framework combines preliminary electrode placement assessment, muscle activity detection, informative EMG feature extraction, weight- and movement-specific reference profile construction, longitudinal progression assessment, and regression-based time prediction. The proposed two-stage activity detection method, which integrates clustering-based activity-region identification with probabilistic onset refinement, achieved an average error of 1.5% and an average temporal deviation of 19 ms under the evaluated conditions. In addition, the preliminary electrode placement assessment showed that positioning the electrode adjacent to the innervation zone produced the highest RMS value among the four tested locations.

Among the five evaluated regression models, Linear Regression provided the best fit to the observed longitudinal data, achieving R^2^ = 0.987 and RMSE = 4.21. The more complex tree-based, kernel-based, and probabilistic models captured the overall progression trend but did not provide an additional predictive advantage within the available dataset. These findings suggest that the selected EMG features may serve as useful sensing indicators for individualized athlete progression assessment and that a simple, interpretable regression model may be sufficient when the observed temporal pattern is predominantly linear.

Nevertheless, the findings should be interpreted cautiously. The electrode placement comparison was limited to one subject, one muscle, and one contraction task, while the regression analysis was based on only six longitudinal observation periods for one representative athlete, with each period represented by the combined 90-dimensional EMG feature vector obtained from all ten movement classes. Consequently, the reported regression performance represents preliminary, athlete-specific goodness of fit rather than evidence of population-level predictive generalization. Future studies should validate the framework using larger longitudinal cohorts, independent athlete datasets, broader muscle groups, and more representative reference profiles. Embedded implementation should also be evaluated before claims regarding real-time wearable or edge-computing deployment can be established.

## Figures and Tables

**Table 1:** General description of the dataset for the research regression model.

Indicator	Value
Sport type	National Wrestling
Number of weight classes	8
Number of EMG features	9
Number of movement classes	10
Number of exercises per class	5
Observation points per class	6
Observation period	1 month
Target variable	Time elapsed since training started (days)

**Table 2:** Placement of sensors in muscles.

No	Channel	Name of Muscle	Electrode Placement
1	1-sensor	Left Erector spinae ileocostalis	Back of left waist
2	2-sensor	Right Erector spinae ileocostalis	Back of right waist
3	3-sensor	Right Rectus femoris	Front of right thigh
4	4-sensor	Left Gastrocnemius lateralis	Back of left calf
5	5-sensor	Right Gastrocnemius lateralis	Back of right calf
6	6-sensor	Left Rectus femoris	Front of left thigh
7	7-sensor	Left Biceps brachii caput brevis	Upper left arm
8	8-sensor	Right Biceps brachii caput brevis	Upper right arm

**Table 3:** Comparison of RMS values depending on the electrode location.

Electrode Placement	RMS Value
Over the IZ	0.0707
Lateral to the IZ	0.0669
Adjacent to the IZ	0.0788
Adjacent to the TZ	0.0523

**Table 4:** Comparison of EMG signal activity threshold detection algorithms.

Algorithm	Average Error (%)	Average Deviation (ms)
Single-threshold method	3.1	32.0
Dual-threshold method	2.6	23.0
Statistical optimal decision (CUSUM/GLRT)	2.1	21.0
SSA-based method	2.0	27.0
Komi algorithm	2.2	24.0
TKEO-based method	1.9	21.0
Proposed method	1.5	19.0

**Table 5:** Summary of feature selection and validation.

Stage	Number of Features	Reduction (%)
Initial feature set	36	0
After Mutual Information	18	50.0
After L1-regularization	9	75.0
Final set validated with SHAP	9	75.0

**Table 6:** Features of the EMG signal.

No	EMG Signal Characteristics	Mathematical Expression
**1**	Simple square integral (SSI)	$SSI=\sum_{i=1}^{N}{\left|x_{i}\right|}^{2}$
**2**	4-Power (4POW)	$4POW=\sum_{i=1}^{N}x_{i}^{4}$
**3**	Mean absolute value (MAV)	$MAV=\frac{1}{N}\sum_{i=1}^{N}\left|x_{i}\right|$
**4**	Waveform length (WL)	$WL=\sum_{i=1}^{N}\left(x_{i}-x_{i-1}\right)$
**5**	Average amplitude change (AAC)	$AAC=\frac{1}{N}\sum_{i=1}^{N-1}\left|x_{i+1}-x_{i}\right|$
**6**	Integrated EMG (IEMG)	$IEMG=\sum_{i=1}^{N-1}\left|x_{i}\right|$
**7**	Root mean square (RMS)	$RMS=\sqrt{\frac{1}{N}\sum_{i=0}^{N-1}{x_{i}}^{2}}$
**8**	Variance of EMG(VAR)	$VAR=\frac{1}{N-1}\sum_{i=1}^{N-1}{x_{i}}^{2}$
**9**	Difference absolute standard deviation (DASDV)	$DASDV=\sqrt{\frac{1}{N-1}\sum_{i=1}^{N-1}{\left(x_{i+1}-x_{i}\right)}^{2}}$

**Table 7:** Range of hyperparameters tested using GridSearchCV.

Model	Parameter	Checked Values
Linear Regression	fit_intercept	True, False
XGBoost	n_estimators	50, 100, 200
	max_depth	2, 3, 4, 5
	learning_rate	0.01, 0.05, 0.1
	subsample	0.8, 1.0
SVR	kernel	linear, rbf
	C	0.1, 1, 10, 100
	epsilon	0.01, 0.1, 0.5
	gamma	scale, auto
CatBoost	depth	2, 3, 4, 5
	learning_rate	0.01, 0.05, 0.1
	iterations	50, 100, 200
Gaussian Process Regression	kernel	RBF, Matern
	alpha	1 × 10^−5^, 1 × 10^−4^, and 1 × 10^−3^
	n_restarts_optimizer	5, 10

**Table 8:** Subject-specific performance of the regression models for a representative athlete from the −73 kg weight category.

Model	RMSE	MAPE	MSE	R^2^
Linear Regression	4.21	0.03	17.72	0.987
XGBoost	6.12	0.05	33.45	0.975
Support Vector Regression (SVR)	7.21	0.06	52.09	0.962
CatBoost	5.89	0.05	34.69	0.978
Gaussian Process Regression (GPR)	6.74	0.06	45.43	0.932

## Data Availability

The data presented in this study are not publicly available because they contain information that could compromise the privacy of the participating athletes and are subject to ethical restrictions. The data may be made available from the corresponding author upon reasonable request.
